# Subdural empyema in patient with SARS-CoV-2 positivity: A rare case report of 3 patients

**DOI:** 10.1016/j.radcr.2024.11.076

**Published:** 2024-12-24

**Authors:** Hamidreza Aghadoost, Ghazaleh Salehabadi, Esmaeil Fakharian

**Affiliations:** a Department of Neurosurgery, Kashan University of Medical Sciences, Kashan, Iran; b Department of Radiology, Iran University of Medical Sciences, Tehran, Iran; c Trauma Research Center, Kashan University of Medical Sciences, Kashan, Iran

**Keywords:** Covid-19, Subdural empyema, Sars-Cov-2, Brain CT scan

## Abstract

Coronavirus disease 2019 (COVID-19) is a viral pandemic and a matter of concern. It also mimics viral pneumonia with cough and fever but also causes severe sequels and various complications. Subdural empyema is a very rare brain infection presenting mostly with fever, weakness, and altered level of consciousness, and has been recently noted as a new complication of COVID-19. This report presents 3 cases of subdural empyema with a prior history of COVID-19. The first case is a 17-year-old female presented with a 1-week history of severe progressive headache, right-sided ptosis, and left-sided hemiplegia with a history of COVID-19 in about 40 days before admission. The second one is a 41-year-old female presented with a 2-week history of fatigue, severe progressive headache which deteriorated while sitting, left tarsal swelling, and left-sided hemiparesis accompanied by drowsiness since 1 day before admission and a history of COVID-19 in about 50 days before admission. The third one is a 47-year-old gentleman with a known case of epilepsy, who presented with a 2-day history of headache and fever and an event of Generalized Tonic Colonic (GTC) seizure on the day of admission. The patient had a history of multiple head traumas during past years, which led to craniectomy, and also a history of COVID-19 about 46 days before admission. Emergent surgery was performed to evacuate empyema, and patients underwent subsequent anti-bacterial treatment. All patients showed clinical improvement. This report highlights a potential link between SARS-COV-2 infection and the patient's development of subdural empyema.

## Introduction

Sars-coV2, which was first discovered in Wuhan, China, in December 2019, soon became a global pandemic and caused a large amount of mortality and morbidity among the human population [1]. Sars-cov2 has been detected to invent different complications to various body organs; the most known one is the respiratory system. As the literature showed, brain infection, which leads to intracranial abscess and subdural empyema, is a rare event caused by Sars-Cov2, mainly presenting with fever, headache, altered level of consciousness, neurologic deficits, etc. [2,3]. Subdural empyema refers to a buildup of pus located between the dura mater and the adjacent arachnoid mater. It accounts for 15%-22% of all intracranial infections. The diagnosis is challenging, and if delayed, the patient would be prone to severe sepsis and ultimate death. The best treatment of subdural empyema is surgical, with the evacuation of the pus and subsequent treatment with antibiotics targeting the bacteria [4]. In September 2021, Charlton et al. reported a case of subdural empyema presenting with unremitting, severe throbbing headaches for 3 weeks in an adolescent who had a history of COVID-19. They highlighted a potential link between SARS-CoV-2 infection and this patient's development of subdural empyema for the first time [4]. Here, we report 3 similar cases, which mainly presented with severe headaches and neurological deficits, with a potential relationship between subdural empyema and SARS-CoV2.

## Case presentation

**Fig. 1:**
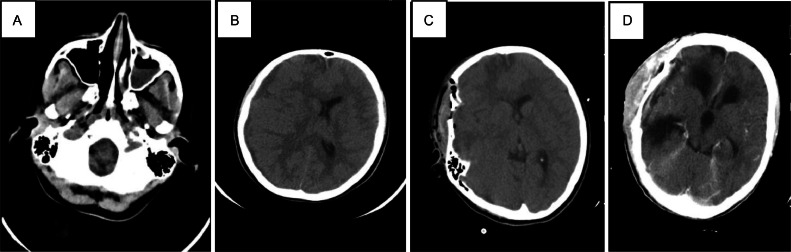
Brain CT scan without contrast (A) air-fluid level in left maxillary sinus suggestive of sinusitis (B) preoperatively showed A hypoattenuating extra-axial fluid collection with a diameter of 7.5 mm over the right hemisphere with a compressive effect on the lateral ventricle and midline shift of 7 mm into the left side was present, considered as subdural empyema. (C) Early postoperative CT scan showed reduced midline shift and drainage of a subdural extra-axial fluid collection, which was diagnosed as subdural empyema. Contrast-enhanced brain CT scan (D) 6 months after surgery showed noncommunicating hydrocephalus.

**Case I:** A 17-year-old Iranian female presented with a 1-week history of severe progressive headache, which was the worst headache the patient had ever experienced, as she mentioned. The headache was mostly sensed in the temporooccipital area and was accompanied by nausea. 3 days before admission, right-sided ptosis and a day before, left-sided hemiparesis, which turned into hemiplegia during a few hours, were also added to the prior complaints. The patient had a history of COVID-19 approximately 40 days prior to admission in July 2023. She experienced a mild disease course consisting mostly of headaches and received no pharmacologic intervention related to the COVID-19 diagnosis. She had no other noticeable past medical history and used no medications at the time of presentation. No history of head trauma was recorded. During the last few weeks, different types of painkillers have been used by the patient, but they were of no help. On clinical examination, she looked comfortable but in mild pain, with a temperature of 37.2 °C, pulse rate of 61 beats per minute, blood pressure of 105/70 mmHg, and respiratory rate of 12 breaths per minute. On admission, neurological examination revealed right eye ptosis, diminished vertical eye movement, deficits in left side trigeminal sensory nerve and left facial hemiparesis, and left-sided hemiplegia (force of left upper and lower limb: 0/5). Leukocyte count, Sedimentation rate, and C-reactive protein values were abnormally high. CT imaging demonstrated opacifications containing air-fluid level in maxillary and frontal sinuses, suggesting acute sinusitis (Fig. 1). A hypoattenuating extra-axial fluid collection with a diameter of 7.5 mm over the right hemisphere with compressive effect on lateral ventricle and midline shift of 7 mm into left side was present, considered as subdural empyema and pachymeningitis probably secondary to acute frontal and maxillary sinusitis (Fig. 1).

The patient underwent a neurosurgery operation. After prep and drape under general anesthesia in a supine position with the head fixed in Mayfield and turned to the left through a large reverse question mark incision, the free bone flap was removed. The Dura opened over the convexity, more than 50 ccs of light green fluid evacuated from cerebral convexity, frontal, and temporal skull base. The cortex had severe reactive fibrosis with some small thrombotic vessels. The medial part of the convexity had an almost normal appearance, and no pus was found there. After copious irrigation and homeostasis, Dura was repaired with a large subperiosteal graft and bone fixed in place by burr-hole plates, 2 eye bars and mini-screws, and a vacuum draining catheter inserted, and the wound closed by layers. Neurosurgeons’ exact diagnosis during the surgery was right frontotemporoparietal subdural empyema. The patient underwent FESS surgery in which antrostomy, anterior and posterior ethmoidectomy, and sphenoidotomy were conducted. The patient underwent an infectious disease consult after the surgery and was discharged with gentamycin, vancomycin, and meropenem. The early postoperative brain CT scan revealed reduced midline shift and drainage of a subdural extra-axial fluid collection, which was diagnosed as subdural empyema (Fig. 1).

During her regular follow-ups at Shahid Beheshti Hospital of Kashan for 6 months, her headache started to improve gradually. A contrast-enhanced brain CT scan was obtained and showed noncommunicating hydrocephalus (Fig. 1). Neurological examination revealed mild left hemiparesis. Inflammatory markers trended down slowly and eventually normalized unless immediate diagnosis and surgical intervention, hydrocephalus, and mild hemiparesis were found as subdural empyema complications during 6 months of follow-up.

**Fig. 2:**
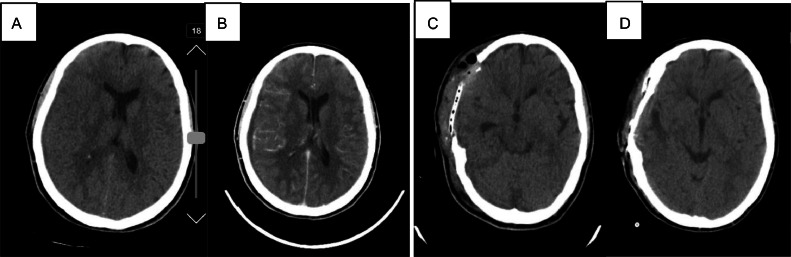
Noncontrast Brain CT scan (A) preoperatively showed a hypodense rim in right frontotemporoparietal with subtle midline shift to the left. Contrast enhanced brain CT scan (B) preoperatively showed a hypodense rim in right frontotemporoparietal with enhancement of adjacent pia matter in favor of a subdural inflammatory collection. (C) Early postoperative CT scan showed right frontotemporoparietal craniectomy, no midline shift with the drainage of a subdural extra-axial fluid collection, which was diagnosed as subdural empyema, (D) One-month postoperatively CT scan showed right frontotemporoparietal cranioplasty with normal brain tissue.

**Case II:** A 41-year-old Iranian female presented with a 2-week history of fatigue, severe progressive headache which deteriorated while sitting, left tarsal swelling, and left-sided hemiparesis accompanied by drowsiness since 1 day prior to admission. The patient had a history of nonspecified psychological problems, which were under control by using medication, and also a history of COVID-19 about 50 days before admission in December 2023. No history of head trauma was recorded. During the last few weeks, different types of painkillers and muscle relaxants have been used by the patient, which didn't lower the headache. On clinical examination, she looked lethargic with a temperature of 40.0 °C, pulse rate of 80 beats per minute, blood pressure of 110/50 mmHg, and respiratory rate of 16 breaths per minute. On admission, the neurological examination could not be done precisely because of the patient's drowsiness, but it revealed motor deficits in left limbs (the patient did not move left limbs at all). Leukocyte count, Sedimentation rate, and C-reactive protein values were abnormally high (ESR=100, CRP=100). Noncontrast Brain CT scan demonstrated a hypodense rim in right frontotemporoparietal with subtle midline shift to the left. Contrast enhanced brain CT scan showed hypodense rim in right frontotemporoparietal with enhancement of adjacent pia matter in favor of a subdural inflammatory collection (Fig. 2).

According to her clinical presentation, prior history of infection with COVID-19, and CT scan findings, she was diagnosed as a case of subdural empyema. She was admitted to the neurosurgery operation room immediately.

After prep and drape under general anesthesia in a supine position with the head in a horseshoe and turned to the left through a large reverse question mark incision, the free bone flap was removed. The Dura opened over the convexity—more than 50 ccs of light-yellow fluid evacuated from cerebral convexity, frontal, and temporal skull base. The cortex had severe reactive fibrosis with some small thrombotic vessels. After copious irrigation and homeostasis, Dura was repaired with a large periosteal graft and a vacuum-draining catheter inserted, and the wound was closed by layers. Neurosurgeons’ exact diagnosis during the surgery was Frontotemporoparietal subdural empyema. The patient underwent an infectious disease consult after the surgery and was discharged with gentamycin, vancomycin, and meropenem. The early post operative CT scan showed right frontotemporoparietal craniectomy, no midline shift with the drainage of a subdural extra-axial fluid collection, which was diagnosed as subdural empyema (Fig. 2).

During his regular follow-ups in Shahid Beheshti hospital in 6 months, his headache started to improve gradually, neurological examination revealed no neurological deficits after the surgery, and inflammatory markers were trending down slowly and eventually normalized.

**Fig. 3:**
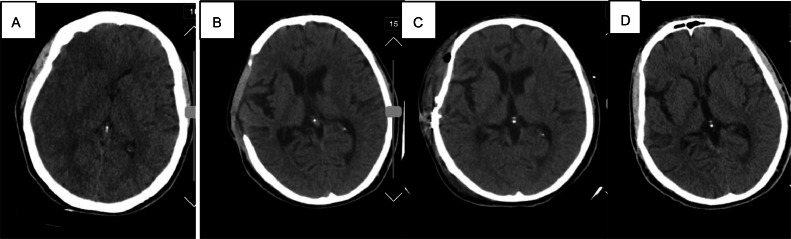
Noncontrast Brain CT scan (A) preoperatively showed a hypodense rim in right frontotemporoparietal with subtle midline shift to the left. (B) Early postoperative CT scan showed right frontotemporoparietal craniectomy, no midline shift with the drainage of a subdural extra-axial fluid collection, which was diagnosed as subdural empyema, (C) One-month postoperatively CT scan showed right frontotemporoparietal cranioplasty with normal brain tissue. (D) Six-month postoperatively CT scan showed normal brain tissue.

**Case III:** A 37-year-old Iranian gentleman who was a known case of epilepsy presented with a 2-day history of headache and fever and an event of Generalized Tonic Colonic (GTC) seizure on the day of admission. The patient had a history of multiple head traumas during past years, and also a history of COVID-19 about 46 days before admission in March 2024. On clinical examination, he looked ill with a temperature of 39.0 °C, pulse rate of 78 beats per minute, blood pressure of 110/65 mmHg, and respiratory rate of 15 breaths per minute. On admission, neurological examination showed no deficits. Leukocyte count, Sedimentation rate, and C-reactive protein values were abnormally high (ESR=100, CRP=250). CT imaging demonstrated a hypodense rim in the right frontotemporoparietal, similar to case I (Fig. 3).

According to his clinical presentation and CT scan findings, he was diagnosed at first as a case of subdural hematoma and was admitted to the neurosurgery operation room.

After prep and drape under general anesthesia in a supine position with the head fixed in Mayfield and turned to the left through a large reverse question mark incision, the free bone flap was removed. Dura opened over the previous frontotemproparietal incision scar. More than 30 ccs of light green fluid evacuated from cerebral convexity, frontal, and temporal skull base. After copious irrigation and homeostasis, Dura was repaired with a large periosteal graft, bone fixed in place by burr-hole plates, a vacuum-draining catheter inserted, and the wound closed by layers. Neurosurgeons’ exact diagnosis during the surgery was right Frontotemporoparietal subdural empyema. The patient was discharged with phenytoin, meropenem, and vancomycin. The early post operative CT scan showed right frontotemporoparietal craniectomy, no midline shift with the drainage of a subdural extra-axial fluid collection, which was diagnosed as subdural empyema (Fig. 3).

During his regular follow-ups at Shahid Beheshti Hospital in 6 months, his headache started to improve gradually. No seizure happened after the surgery, and inflammatory markers trended down slowly and eventually normalized.

## Discussion

Descriptions of the mentioned cases, suggest the idea of temporal relationship between SARS-CoV2 infection and the development of subdural empyema. Subdural empyema is a very rare brain infection mostly happen in patients with a history of chronic sinusitis, otitis or mastoiditis [5]. Previous studies declared that headache and fever are the most important and common signs of subdural empyema or brain abscess, but we found neurological deficits such as ptosis and hemiplegia as very important and noticeable signs of subdural empyema in our patients.

Since SARS-CoV2 infection is a matter of concern worldwide, it is essential to know its hazardous complications. Table 1 summarizes the presentations of previous case reports, which are in line with our study and develop the idea of a relationship between SARS-CoV2 and subdural empyema.

**Table 1:** Previous case reports of subdural empyema after COVID-19 infection.

First author	Year	Country	Case-presentation	Imaging findings	Treatment	Outcome
Megan Charlton [4]	2021	USA	65-year-old male with a recent history of SARS-CoV-2 infection who presented with 3 weeks of escalating headache.	CT scan showed a hypoattenuating extra-axial fluid collection over the left anterior frontal convexity. MRI showed extra-axial collection demonstrating restricted diffusion identified along the left frontal lobe.	He subsequently underwent 2 craniectomies, which resulted in eradication of the abscess and clinical improvement.	After 25 days spent in rehabilitation, presented to the emergency department with calf pain, weakness, confusion, and an episode of presyncope and was diagnosed with bilateral and saddle pulmonary embolisms.

Kaisar Haroon [6]	2021	Bangladesh	A 13-year-old girl suspected to Covid-19 whose RT-PCR was negative at first, presented with the headache, fever, convulsion once, blurring of vision and vomiting.	Her MRI scan of brain with contrast showed acute subdural empyema involving the interhemispheric fissure along the parafalcine space.	She underwent a frontoparietal craniotomy on the right side. dura was opened and some pus had escaped. The parafalcine space was approached and all pus was evacuated from the interhemispheric space.	The bone was replaced after 3 months and, after the second surgery, she developed symptoms of fever, malaise, body ache and rhinorrhea and therefore a second sample for RT-PCR for COVID 19 was sent. This time report was positive for COVID 1 9.

Lucas Crociati Meguins [7]	2021	Brazil	A 49-year-old man with COVID-19 developed pneumonia. Brain CT was performed with evidence of communicating hydrocephalus. External ventricular shunt (EVD) was implant with intraoperative cerebrospinal fluid suggestive of meningitis Twenty days after EVD, meningitis treatment was finished and with 2 negative cultures, conversion to ventriculoperitoneal shunt was performed. In the following week, quadriplegia and absence of spontaneous respiratory movement were evidenced, just maintaining head movement.	Brain MRI was performed with a diagnosis of ventriculitis associated with pus collections on the IV ventricle.	the patient underwent microsurgical drainage removal of the shunt, with a positive intraventricular collection culture for *Klebsiella pneumoniae* carbapenemase and multidrug-resistant *Pseudomonas aeruginosa*, without improvement in the neurological condition.	After 14 weeks of hospitalization, the patient died.

Vladimir A Ljubimov [8]	2022	USA	15-year-old boy presented with left sided weakness, frontal sinusitis while having active COVID-19 infection.	MRI showed evidence of the subdural empyema, infected fluid collection in the right frontal sinus	Emergent surgery was performed for evacuation of empyema and sinus debridement.	Over the next 2 weeks, the patient continued to make improvements in motor strength and sensation. With intensive physical therapy, he regained complete strength of his left side and was able to walk and use his left arm functionally. MRI of the brain at 6 months after surgery showed no evidence of recurrent infection intracranially or in the sinuses.
Cian Duggan [9]	2022	Ireland	An 11-year-old male presented with pyrexia for over 5 days, headache, myalgia, anorexia, malaise, nonpurulent conjunctivitis, subjective photophobia, vomiting and decreased oral intake, with a normal neurological examination on a background of Covid-19 diagnosed 7 days previously to first admission.	His CT brain demonstrated a left subdural empyema. 6mm in maximal depth overlying left cerebral hemisphere and extends medially along the falx. Significant mass effect with approximately 8mm midline shift and effacement of the left lateral ventricle.	surgery was performed for evacuation of empyema. A culture of left parietal pus obtained intraoperatively grew Streptococcus Constellatus.	intracranial drain removed after 20 days. He currently had a mild right hemiparesis and mild dysphasia.

Uğur Yazar [10]	2022	Turkey	15-year-old and 12-year-old female children with limitation of eye in the outward gaze, impaired speech, drowsiness, fever, vomiting and they also were tested positive for COVID-19.	MRI showed subdural empyema along a left frontotemporoparietal and right tentorium with midline shift in case I. Contrast enhanced MRI showed interhemispheric subdural empyema on vertex level in case II.	surgical intervention was done to relieve intracranial pressure and drain pus after receiving broad spectrum antibiotics treatments.	Eventually, patients became neurologically intact.

Christopher S Hong [11]	2023	USA	20-year-old otherwise healthy immunocompetent male who was recently diagnosed with COVID-19 with respiratory symptoms and unresponsive fever.	MRI demonstrated pansinusitis, diffuse pachymeningeal and leptomeningeal enhancement, and a left frontoparietal diffusion-restricting and enhancing subdural collection, concerning for empyema.	The patient underwent left decompressive hemicraniectomy for subdural empyema evacuation, which revealed pus above the dura, secondary to a bony defect in the frontal sinus, which was repaired. Gross subdural pus was found overlying the brain upon dural opening. Subsequently, he underwent bilateral complete endoscopic sinus surgery, which redemonstrated pus emanating from the frontal sinus	At the time of the last follow-up, 4 months after initial presentation, he had completed his antifungal therapy, was in neurologically intact condition, and had undergone a successful left cranioplasty procedure to address his existing bony skull defect.

Aldo Jose Ferreira da Silva [12]	2023	Brazil	12-year-old female, with a history of headache and vomiting, without fever, with progressive worsening and coma. Laboratory tests showed positive SARS-CoV-2 PCR RNA.	Noncontrast-enhanced cranial tomography showed a right fronto-temporo-parietal cortical hypodense area with significant midline shift	A decompressive craniectomy was performed with drainage of extensive subdural empyema	Not written.

The very first case of this presentation was admitted to our department in July 2023, and the other ones in December 2023 and March 2024, respectively. To our knowledge, during a period of 8 months time, multiple cases of subdural empyema with a history of COVID-19 have not been reported yet. Subdural empyema is a medical and surgical emergency. Time is of the utmost importance when this pathology is encountered, and early recognition and management are required. All 3 patients presented in this case report immediately underwent surgical intervention after clinical suspicion and obtaining a brain CT scan. They all recovered well after 6 months of follow-up, and no neurologic deficit or other medical complications were noted.

This report highlights a potential link between SARS-COV2 infection and the patient's development of subdural empyema. Strong clinical suspicion is essential to accurately diagnose and treat patients who may experience this complication of SARS-CoV-2 infection.

## Conclusion

Subdural empyema is a life-threatening emergency demands immediate treatment. Although it is rare to occur as the initial presentation of an underlying condition like Sars-Cov-2, health care providers must maintain a high index of suspicion to avoid missing this critical diagnosis and ensure timely intervention. The case also encourages further research and documentation of similar rare cases to increase understanding and improve the clinical outcomes of such rare cases.

## Author contributions

Conceptualization: E.F, H.A; Writing—original draft preparation: H.A, G.S; Writing—review and editing: E.F, G.S,H.A.

## Patient consent

Signed consent was taken from the patients which can be made available upon reasonable request from the lead author.
